# Supplementary figures and images for: Capture of fusion-intermediate conformations of SARS-CoV-2 spike requires receptor binding and cleavage at either the S1/S2 or S2’ site
Source: PLoS Pathog. 2025 Apr 8;21(4):e1012808. doi: 10.1371/journal.ppat.1012808 (PMC12011290; doi:10.1371/journal.ppat.1012808)

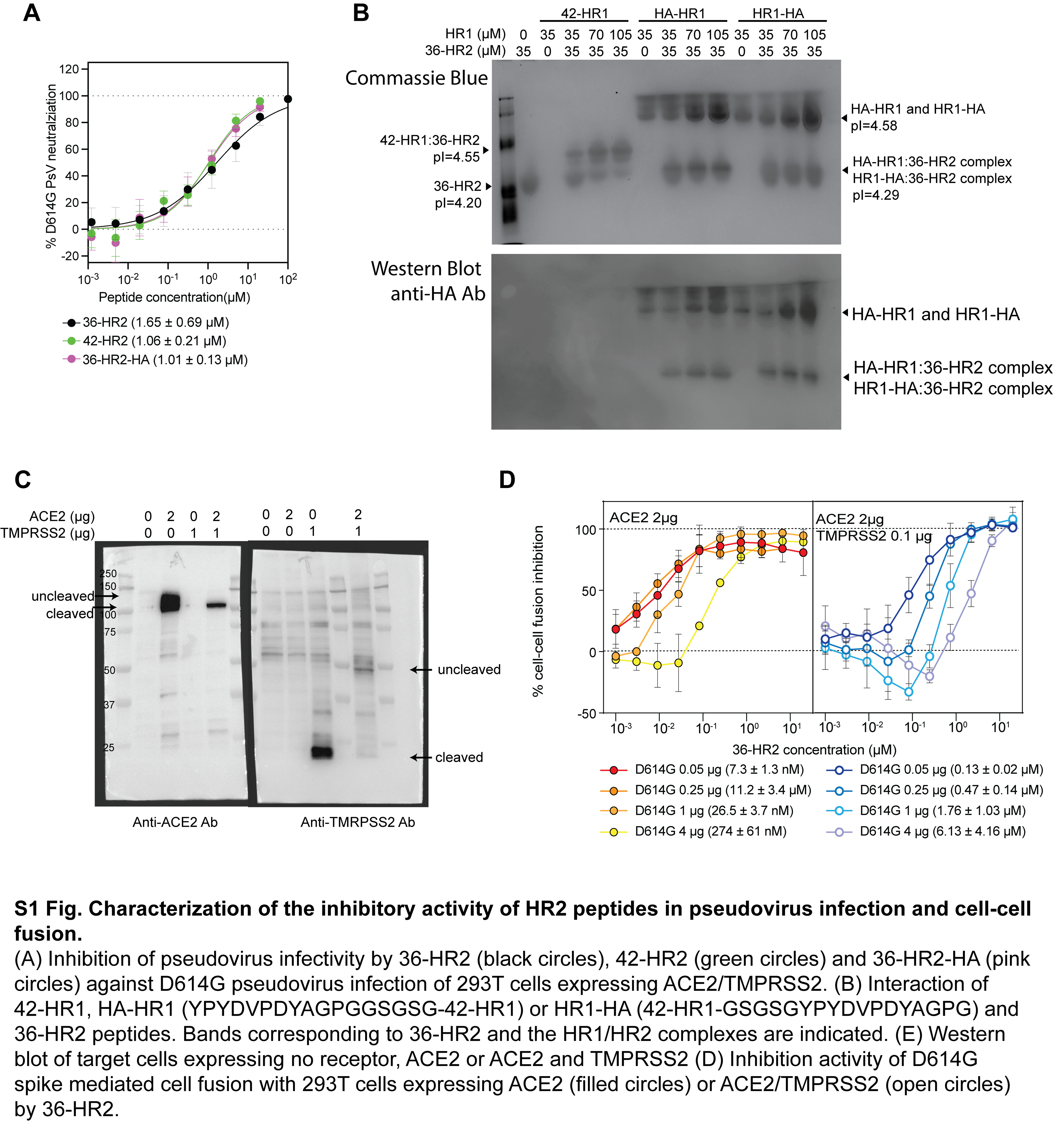

Supplement: S1 Fig — (A) Inhibition of pseudovirus infectivity by the 36-HR2 (black circles), 42-HR2 (green circles) and 36-HR2-HA (pink circles) peptides against D614G pseudovirus infection of 293T cells expressing ACE2/TMPRSS2. (B) Interaction of 42-HR1, HA-HR1 (YPYDVPDYAGPGGSGSG-42-HR1) or HR1-HA (42-HR1-GSGSGYPYDVPDYAGPG) and 36-HR2 peptides. Bands corresponding to 36-HR2 and the HR1/HR2 complexes are indicated. (E) Western blot of control target cells without receptor, ACE2 or ACE2 and TMPRSS2 (D) Inhibition activity of D614G spike mediated cell fusion with 293T cells expressing ACE2 (filled circles) or ACE2/TMPRSS2 (open circles) by 36-HR2. (TIF) [file ppat.1012808.s001.tif]

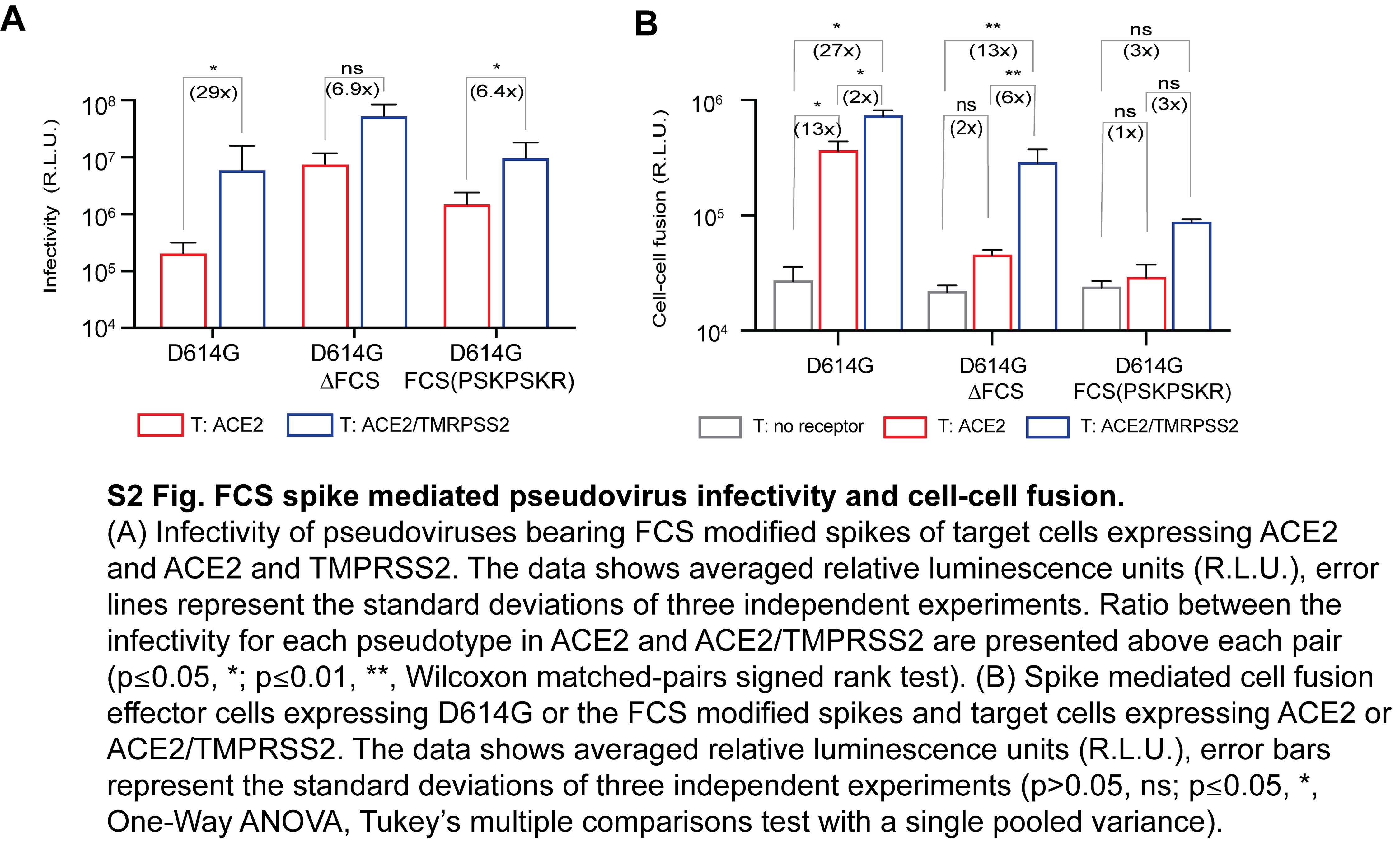

Supplement: S2 Fig — (A) Infectivity of pseudoviruses bearing FCS modified spikes of target cells expressing ACE2 and ACE2 and TMPRSS2. The data shows averaged relative luminescence units (R.L.U.), error lines represent the standard deviations of three independent experiments. Ratio between the infectivity for each pseudotype in ACE2 and ACE2/TMPRSS2 are presented above each pair (p≤0.05, *; p≤0.01, **, Wilcoxon matched-pairs signed rank test). (B) Spike mediated cell fusion effector cells expressing D614G or the FCS modified spikes and target cells expressing ACE2 or ACE2/TMPRSS2. The data shows averaged relative luminescence units (R.L.U.), error bars represent the standard deviations of three independent experiments (p>0.05, ns; p≤0.05, *, One-Way ANOVA, Tukey’s multiple comparisons test with a single pooled variance). (TIF) [file ppat.1012808.s002.tif]

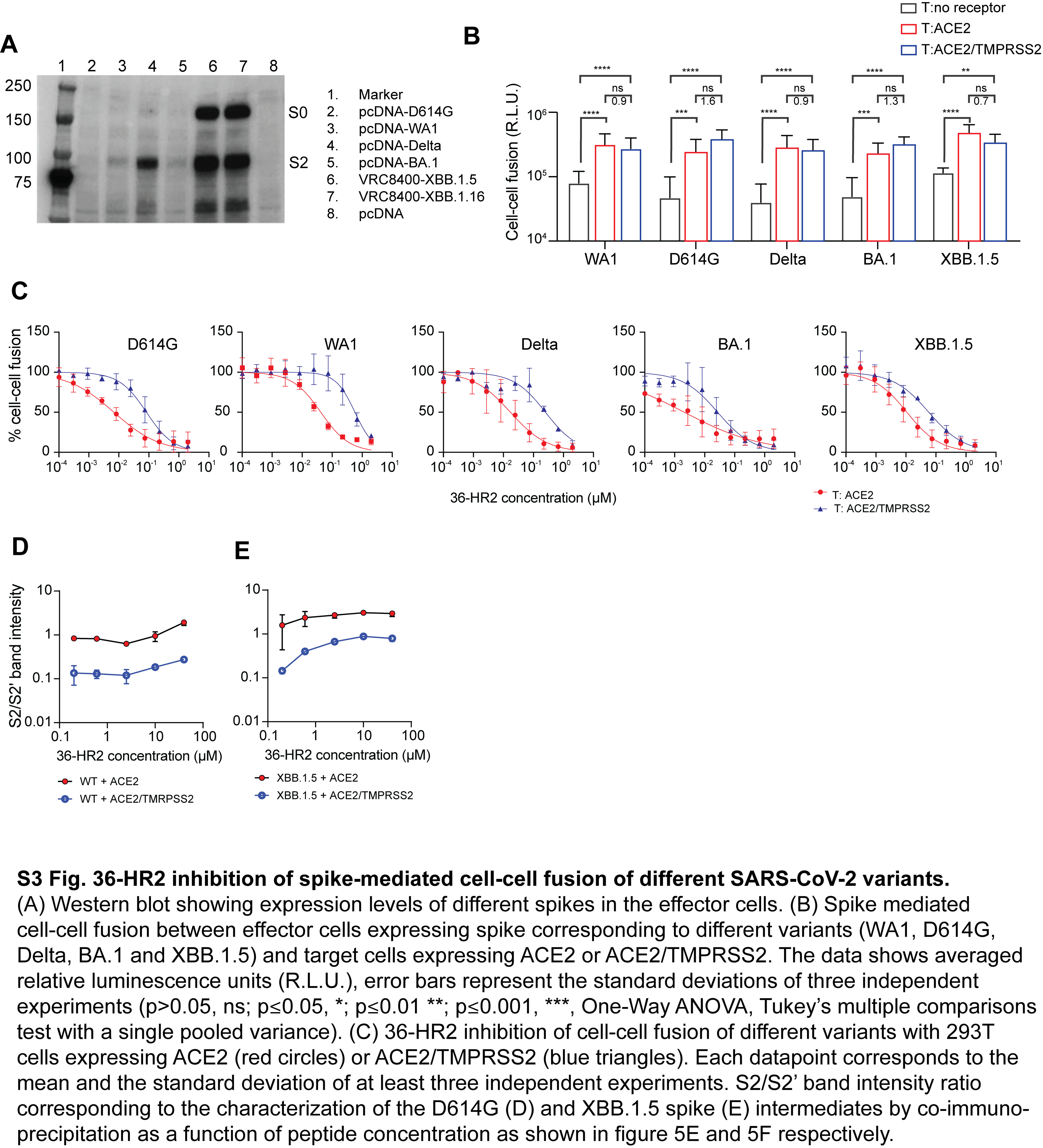

Supplement: S3 Fig — (A) Western blot showing expression levels of different spikes in the effector cells. (B) Spike mediated cell-cell fusion between effector cells expressing spike corresponding to different variants (WA1, D614G, Delta, BA.1 and XBB.1.5) and target cells expressing ACE2 or ACE2/TMPRSS2. The data shows averaged relative luminescence units (R.L.U.), error bars represent the standard deviations of three independent experiments (p>0.05, ns; p≤0.05, *; p≤0.01 **; p≤0.001, ***, One-Way ANOVA, Tukey’s multiple comparisons test with a single pooled variance). (C) 36-HR2 inhibition of cell-cell fusion of different variants with 293T cells expressing ACE2 (red circles) or ACE2/TMPRSS2 (blue triangles). Each datapoint corresponds to the mean and the standard deviation of at least three independent experiments. S2/S2’ band intensity ratio corresponding to the characterization of the D614G (D) and XBB.1.5 spike (E) intermediates by co-immunoprecipitation as a function of peptide concentration as shown in Fig 5E and 5F respectively. (TIF) [file ppat.1012808.s003.tif]
